# Prevalence of diabetes in Northern African countries: the case of Tunisia

**DOI:** 10.1186/1471-2458-14-86

**Published:** 2014-01-28

**Authors:** Habiba Ben Romdhane, Samir Ben Ali, Wafa Aissi, Pierre Traissac, Hajer Aounallah-Skhiri, Souha Bougatef, Bernard Maire, Francis Delpeuch, Noureddine Achour

**Affiliations:** 1 Cardiovascular Epidemiology and Prevention Research Laboratory, Faculty of Medicine, 15 rue Djebel Akdhar-La Rabta-1007 Bab Saâdoun, Tunis, Tunisia; 2 IRD (Institut de Recherche pour le Développement), UMR 204 NUTRIPASS, IRD-UM1-UM2, 911, av. Agropolis, 34394 Montpellier, France; 3 National Public Health Institute, 7 rue Khartoum, Tunis 1005, Tunisia

**Keywords:** Prevalence, Type 2 Diabetes, Diagnosed, Undiagnosed, Impaired fasting glucose, Sociodemographic factors

## Abstract

**Background:**

Although diabetes is recognized as an emerging disease in African and Middle East, few population-based surveys have been conducted in this region. We performed a national survey to estimate the prevalence of type 2 diabetes (T2D) and to evaluate the relationship between this diagnosis, demographic and socioeconomic variables.

**Methods:**

The study was conducted on a random sample of 6580 households (940 in each region). 7700 subjects adults 35–70 years old were included in the analyses. T2D was assessed on the basis of a questionnaire and fasting blood glucose level according to the WHO criteria. Access to health care and diabetes management were also assessed.

**Results:**

Overall, the prevalence of T2D was 15.1%. There were sharp urban vs. rural contrasts, the prevalence of diabetes being twice higher in urban area. However, the ratio urban/rural varied from 3 in the less developed region to 1.6 in the most developed ones. A sharp increase of prevalence of T2D with economic level of the household was observed. For both genders those with a family history of T2D were much more at risk of T2D than those without. Awareness increase with age, economic level and were higher amongst those with family history of T2D. Drugs were supplied by primary health care centers for 57.7% with a difference according to gender, 48.9% for men vs. 66.0% women (*p* < 0.001) and area, 53.3% on urban area vs. 75.2% on rural one (*p* < 0.001).

**Conclusions:**

Through its capacity to provide the data on the burden of diabetes in the context of the epidemiological transition that North Africa is facing, this survey will not only be valuable source for health care planners in Tunisia, but will also serve as an important research for the study of diabetes in the region where data is scarce. In this context, NCDs emerge as an intersectoral challenge and their social determinants requiring social, food and environmental health policy.

## Background

The Eastern Mediterranean Region (EMR) has been recognized as a growing hot spot for Cardiovascular Diseases (CVD) and type2 diabetes. Projections of the growing burden exceed those of most other regions. About 47% of the region’s current burden of disease is due to non-communicable diseases (NCDs), and the Global Burden of Disease project have estimated that this proportion will rise to about 60% by the year 2020 [1]. Rates of coronary heart disease (CHD) will have increased by 160% in the region of Middle East and North Africa [2]. Tunisia is a Northern African country, with a population of about ten million, is typical amongst emerging South and East Mediterranean countries, having recently undergone a rapid economic development and is currently ranked 98th out of 177 on the Human Development Index composite scale in 2009 [3].

Tunisia has experienced a crucial demographic transition, reflecting a sustained and integrated economical, social and health development. The global fertility rate is about 2 and the population is still young, with 24% under 15 years. However the population aged over 65 years is rapidly increasing, already exceeding 6% [4]. Now, Tunisia is facing a rapidly growing burden of NCDs and CVDs are the leading causes of death accounting for almost 30%, 70% of those are CHD death [5,6].

Trends in conventional cardiovascular risk factors are well documented in Tunisia. Levels are dramatically high, especially in the coastal area [7,8]. According to the last survey conducted on 1996, T2D prevalence was 9.9% [9].

This study aims to estimate the prevalence, awareness, and treatment of T2D at the national level using relevant quantitative measures and to assess, through appropriate modeling, variation according to environment and socio-economic characteristics.

## Method

### Study area

Tunisia is a North African country, situated between Algeria at west and Libya at east. With about 163 000 km^2^, it is the smallest in North Africa. It features sharp geographical contrasts such as a long Mediterranean coastline in the north and the east but more mountainous and remote regions on the west. With10 million inhabitants (of which about two third are urban). Life expectancy at birth of 74 years on man and 78 on women, In the last decades, Tunisia has undergone a steady development in the context of a market-oriented economy, with significant agricultural, mining, tourism, and manufacturing sectors. Gross domestic product per capita 8.258 on PPP US$ ranked before Algeria and Morocco and Human Development Index at 0.712, one of the best in the region.

But this level of human development is unevenly distributed, higher in the main cities and in the eastern coastal regions due to prosperous industrial and tourist activities, with the District of Tunis (the capital) in the North East being the most urbanized and developed.

### Target population and sampling

Non-institutionalized adults aged 35 to 70 years residing in private dwellings in each of the 7 administrative regions of the country included in the survey if they had resided permanently at the address prior to the survey.

### Sampling

The national cross-sectional survey was carried out from April to September, 2005. The target population was all Tunisian adults aged 35 to 70 years. It was based on a national stratified three stage cluster sample [10] of subjects; the sampling frame was derived by the Tunisian National Institute of Statistics from the database of the most recent census of the population carried out in 2004 [11]. Stratification was according to the seven administrative regions which divide Tunisia, each region being a stratum. The first and second stage of random selection were performed using the national census database: in each of the 7 strata, at the first stage 47 census districts were randomly selected, with a probability proportional to size in number of eligible households (i.e. featuring at least then one 35–70 years subject). At the second stage, 25 eligible households were randomly sampled in each district. The third stage of selection was performed during the implementation of the field survey: in each household subject from the targeted age was included in the survey.

The sample size was selected based on precision of estimates to identify national diabetes prevalence of 10.0% (an estimation based on results of previous regional surveys). As a secondary objective of the study was to deliver useful regional-specific prevalence estimates, the sampling frame was stratified at the region level. With very little loss of efficiency, an accurate national estimate can be obtained from weighted samples of equal size from the seven regions of the countries. Accounting for the clustering of the survey design, a sample size of 6,300 (900 per region) was predicted to provide 95% confidence intervals of 9.0% – 10.1%, around a diabetes estimate of 10.0%. However, we decided to include 1200 adults per region to enhance the power to describe the prevalence according to various variables.

The total sample size was 8,400 adults from the target population corresponding to 6000 household as according to the last national census, 1.44 subjects per households are eligible (2004).

### Household census and interview

Following meeting with the community representatives, all private dwellings within the sampled cluster received a hand-delivered letter informing residents about the survey and advising that a health professional team (physician, nutritionist and nurse) would visit to conduct the household interview. A confidential letter to the health center was delivered to interviewed when a disease is diagnosed (hypertension, diabetes) or when the disease was not controlled.

In order to obtain a personal interview with all eligible household members, interviewers made appointments to visit as often as was necessary to the household.

### Measurements

#### Socio-economic and demographic variables

Data on age, gender, marital status, level of education and professional occupation (and for women, parity and menopause) of the subject were collected by interview. Levels of education and professional occupation were defined according to the classification of National Tunisian Institute of Statistics [4].

An asset based proxy index for the economic level of the household was derived from multivariate analysis of relevant items in the Tunisian context.

The study protocol was carried out according to the Declaration of Helsinki and has been ethically approved by the Tunisian Ministry of Health and the Tunisian National Council of Statistics (visa n°5/2005). All participants gave their free informed consent, after being thoroughly informed on the purpose, requirement and procedures of the survey.

Blood samples were collected after a 12-h overnight fast. FPG was determined by the glucose oxidase enzymatic method using a Beckman reagent Kit on a Beckman SYNCHRON CX7 analyzer. T2D was determined by FPG levels, self-report, and antidiabetic medication status. T2D was defined using the World Health organization (WHO) criteria [12] as FPG ≥ 6.1 mmol/L, or confirmed medication usage from the medication inventory, or self reported use of antidiabetic medications within the past 2 weeks of the examination, or self-reported diabetes diagnosis. Impaired fasting glucose (IFG) was defined as FPG ≥ 5.6 mmol/L but < 6.1 mmol/L. Participants who answered “yes” to the interview question “have you ever been told by a doctor or health professional that you have T2D?” were identified as having diagnosed diabetes. The family history of diabetes, age of diagnosis, and antidiabetic treatment type and duration were obtained in the interview. Individuals with diagnosed diabetes were classified as treated if they reported taking antidiabetic medication within the 2 weeks before the examination, or if inventory and classification of their medications at the examination documented use of antidiabetic medication.

#### Data management and statistical analysis

Epidata software, version 3.1 was used for data entry and validation and Stata 12 for data management and analysis [13,14].

Most analyses were performed separately for each gender, except when otherwise stated. Beyond descriptive analyses, the strength of associations (crude or adjusted) of environmental and socio-demographic factors with diabetes status variables was assessed - for binary response variables (diabetes yes/no, previously undiagnosed diabetes yes/no untreated diabetes yes/no) by Odds-Ratios (OR) estimated in logistic regression models; - for the three category FPG status response variable (“diabetes”, “IFG”, “normal”) by multinomial logistic models.

Firstly, descriptive comparisons between genders, areas and regions were performed, and then models including interaction terms for gender × area or region and/or age × area or region within gender were used to assess modifying effects when relevant. Secondly, based on the complete case analysis subsample, associations between environmental factors (milieu, region), demographic (age, parity), familial (history of diabetes), socio-economic factors and diabetes status (yes/no) were assessed within genders. Crude associations were first assessed using univariate models (models 0), then associations where adjusted for demographic and socio-economic factors only (models 1) and a final model (model 2) enabled estimation of associations of diabetes status with all variables adjusted for one another. Analogous analyses were performed for undiagnosed diabetes (yes/no) and untreated diabetes (yes/no) but with data from both genders pooled. Effect of adjustments on strength of associations was assessed by computing confounding ratios: CR = (Adjusted OR – unadjusted OR)/adjusted OR.

The sampling design i.e. stratification, clustering and sampling weights (accounting for differential probabilities of selection and also a post-stratification on gender, age and urban vs. rural) was taken into account in all estimates and analyses using the specific *svy* series of Stata commands. For multivariate analyses, the “complete-case” analysis approach was used to deal with item non response. The type I error risk was set at 0.05 for all analyses. Results are given as estimates and 0.95 confidence interval (in brackets) when relevant.

### Ethics

The protocol was approved by the Ethics Committee of the National the Tunisian National Council of Statistics (visa nu5/2005). All participants gave their free informed consent and data was analyzed anonymously.

## Results

### Survey response

In total, 8400 adults aged 35–70 years old were to be included in the 47 census districts selected per region. Of these, 393 (4, 6%) were classified as non-contacts. Reasons for non-contact (and hence non-participation) in the household interview included the householders not being contactable despite several attempts, no access gained to the residence (e.g. refusal of the householder).

Finally, after exclusion of individuals with missing data on fasting plasma glucose (FPG) or diabetes data 7700 subjects (3225 men and 4475 women) were included in the analyses.

### Socio-demographic characteristics

Socio-demographics characteristics are presented in (Table 1). The post-stratified estimate of the proportion of women was close to half but there was nonetheless a lower response rate for men. Mean age was 49.0 (0.2) year. Almost a quarter of the subjects declared a family history of T2D. Level of education and professional activity, were markedly different between genders. Urban vs. rural areas featured marked contrasts regarding, mean household level proxy (60.9 vs. 35.0, *p* < 0.001), proportion of no schooling (12.5% vs. 35.9%, *p* < 0.001) for men and (37.8% vs. 73.4%, p < 0.001) for women, family history of T2D (30.7% vs. 17.2%, *p* < 0.001), and parity (4.3 vs. 5.5, *p* < 0.001). The 7 regions featured even sharper contrasts (detailed data not shown): e.g. regarding proportion of urban population (34.2% to 92.6%, *p* < 0.001), mean household level proxy (36.5 to 67.4, *p* < 0.001), proportion with no schooling (11.1% to. 36.7%, *p* < 0.001) for men and (31.8% to 73.5%, *p* < 0.001) for women, family history of T2D (15.0% to 32.7%, *p* < 0.001); parity (3.8 to 5.8, *p* < 0.001).

**Table 1:** Distribution of environmental and socio demographic factors among 35–70 years Tunisian adults, by gender (n = 7700)

	**Men**		**Women**	
	**n**	**Weighted %**	**n**	**Weighted %**
	3225	48.4	4475	51.6
**Environment**				
**Area**	3225		4475	
Urban	1870	67.7	2527	66.6
Rural	1355	32.3	1948	33.4
**Region**	3225		4475	
District of Tunis	401	25.8	603	25.3
North East	505	14.3	544	13.7
North West	484	13.1	687	13.5
Centre East	483	21.4	599	21.5
Centre West	510	11.9	669	12.2
South East	427	8.3	681	8.5
South West	415	5.2	692	5.3
**Familial & demographic factors**				
**Familial history T2D**	3211		4457	
Yes	717	25.2	1096	27.1
No	2494	74.8	3361	72.8
**Age (year)**	3225		4475	
35-39	580	18.4	839	21.0
40-44	716	23.6	880	22.1
45-49	583	18.2	827	16.5
50-54	435	13.5	671	14.1
55-59	306	10.1	470	10.3
60-64	247	7.1	335	7.4
65+	358	9.1	453	8.6
**Parity**			4192	
0-3	-	-	1306	37.4
4-5	-	-	1289	31.2
6+	-	-	1597	31.4
**Socio-economic factors**				
**Economic level of the household**	3056		4233	
1st quintile	687	18.5	1074	20.9
2nd quintile	669	18.5	1036	21.2
3rd quintile	609	18.7	886	19.5
4th quintile	606	22.1	705	19.0
5th quintile	485	22.2	532	19.4
**Level of education of head of household**	3186		4386	
No formal schooling	887	23.5	1996	38.8
Primary school	1327	41.4	1557	36.8
Secondary	722	24.3	662	17.3
University	250	10.8	201	7.2
**Level of education**	3213		4465	
No formal schooling	793	20.1	2265	49.7
Primary school	1290	38.9	1322	31.0
Secondary	864	29.6	493	15.4
University	266	11.5	97	3.8
**Professional activity**	2767		2945	
Unemployed/retired	94	3.2	2304	71.0
Employee/worker	1763	61.7	384	16.9
Intermediate	466	18.0	178	7.9
Upper	444	17.1	79	4.2

### Prevalence of T2D and IFG

Overall, the prevalence of T2D and IFG was respectively 15.1% and 5.9% with no major gender difference e.g. for T2D 16.1% for men vs. 14.1% for women. The overall prevalence of combined T2D and IFG was 20.9%, higher in men than in women (22.3% vs. 19.7%, *p* = 0.025) (detailed data not shown). There were sharp urban vs. rural contrasts (*p* < 0.001) mostly due to the prevalence of T2D being twice higher in urban vs. rural areas (17.7% vs. 9.7%), the difference being milder for IFG. But those urban vs. rural contrasts regarding T2D and IFG were not sex specific (milieu area × gender interaction p = 0.85), e.g. the urban vs. rural contrast for T2D being very similar for men [OR (95% CI), 2.1 (1.5-2.8)] and women [OR (95% CI), 1.9 (1.5-2.5)].

Among people with T2D, the percentage of undiagnosed was almost 51.1%; respectively 54.8% on men vs. 47.2% on women, *p* = 0.032. There was no difference according to area and region. Among previously diagnosed for T2D, only 11.7% declared not undergoing treatment. There was no difference according to gender and area (Table 2).

**Table 2:** Distribution of IFG and type 2 diabetes, undiagnosed diabetes and untreated diabetes overall, by gender and by milieu (n = 7700)

	**Urban**		**Rural**		**Urban vs. Rural**	**National**	
	**n**	**%**^**1**^	**n**	**%**^**1**^	***p*-value**^**2**^	**n**	**%**^**1**^
**Men**							
T2D	345	18.9	138	10.2	< 0.001	519	16.1
Impaired FG	121	6.5	76	5.5		32	6.2
Undiagnosed T2D^3^	329	53.3	135	60.7	0.23	464	54.8
Untreated T2D^4^	146	9.9	55	8.4	0.75	201	9.6
**Women**							
T2D	419	16.6	179	9.2	< 0.001	631	14.1
Impaired FG	154	6.1	89	4.6		251	5.6
Undiagnosed T2D^3^	421	47.2	172	47.2	0.99	593	47.2
Untreated T2D^4^	226	13.7	90	12.9	0.87	316	13.5
**Both genders**							
T2D	778	17.7	320	9.7	< 0.001	116	15.1
Impaired FG	277	6.3	165	5.0		454	5.9
Undiagnosed T2D^3^	750	50.4	307	53.9	0.43	1057	51.1
Untreated T2D^4^	372	11.9	145	11.0	0.81	517	11.7

1- Weighted percentage.

2- *p*-value for urban vs. rural.

3- % of diabetes cases not diagnosed prior to the survey: n is total number of observed diabetes cases.

4- % of untreated diabetes cases: n is number of diabetes cases diagnosed prior to the survey and for which treatment information (regarding insulin and/or oral hypoglycemic drugs) was available.

**Figure 1:**
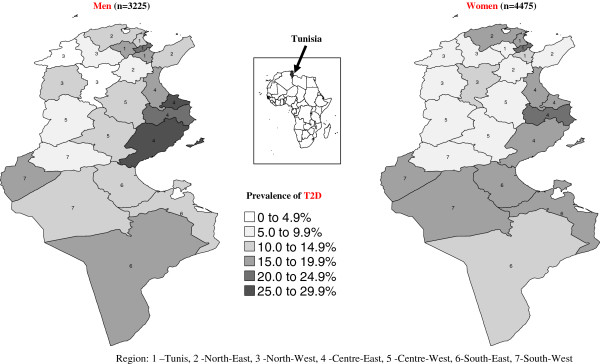
Geographic distribution (governorates) of prevalence of type 2 diabetes by gender (n = 7700).

There were marked differences (*p* < 0.001) between the seven regions regarding prevalence of T2D and IFG (detailed data not shown): T2D prevalence was above the national average in the South-East, District of Tunis and Centre-East regions (respectively 15.9%, 17.9% and 21.1%), almost twice higher than in the North-West and Centre West (respectively 8.2% and 9.6%), with intermediate levels for the North-East and South-West (respectively 12.0% and 12.7%). The geographic distribution was sex specific (region × gender, *p* = 0.003): region rankings based on prevalence of T2D differed according to gender (Figure 1).

**Figure 2:**
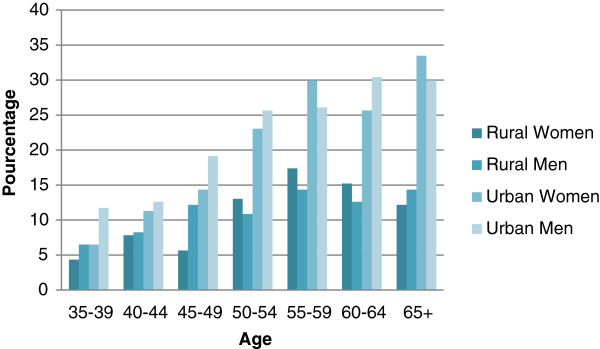
Prevalence of type 2 diabetes by age, gender and urban vs. rural (n = 7700).

For both men and women, the prevalence of T2D increased significantly with age, but the increase was different according to residence areas (Figure 2). In men, it was higher in urban area (11.2% for the age group 35–39 years to 30.5% for the age group 60–64 years) vs. respectively 5.4% and 10% in rural area. In women, the prevalence increased from 5.5% for the age group 35–39 years to 34.0% for the age group 60–64 years in urban area vs. 3.7% to 17.3% for the age group 55–59 years for rural area with is a curve in rural area from 60 years.

Adjusted for demographic and socioeconomic factors (model 1 in Table 3), the urban vs. rural contrast were less marked than for unadjusted one (model 0 in Table 3), more for men (CR = 27%) than for women (CR = 12%). For men inter-regional contrasts (model 0) were stronger than urban vs. rural ones; except for Tunis area (CR = 30%), mediating effect of socio-demographic factors was generally mild (model 1). Some residual regional differences persist even when taking into account urbanization and family history of T2D (model 2). For women, inter-regional contrasts were similar to urban vs. rural ones, but less accounted for by urbanization and socio-demographic or family history of T2D. For both genders those with a family history of T2D were much more at risk of T2D than those without (OR = 2.5 for men and OR = 2.3 for women), and this association did persist after adjustment for all the other co-variables.

**Table 3:** Association of environmental and socio-demographic variables with type 2 diabetes, by gender (n = 6908)

	**Men (n = 3013)**								**Women (n = 3895)**							
		**Crude associations**			**Adjusted associations**		**Adjusted associations**				**Crude associations**		**Adjusted associations**		**Adjusted associations**	
		**(Model 0)**^**1**^			**(Model 1)**^**2**^		**(Model 2)**^**3**^				**(Model 0)**^**1**^		**(Model 1)**^**2**^		**(Model 2)**^**3**^	
	**n**	**%**^**4**^	**OR**^**5**^	**C.I.**^**6**^	**OR**^**5**^	**C.I.**^**6**^	**OR**^**5**^	**C.I.**^**6**^	**n**	**%**^**4**^	**OR**^**5**^	**C.I.**^**6**^	**OR**^**5**^	**C.I.**^**6**^	**OR**^**5**^	**C.I.**^**6**^
**Environment**																
**Area**	3013	*p* < 0.001			*p* = 0.030		*p* = 0.11		3895	*p* < 0.001			*p* = 0.0067		*p* = 0.076	
Urban	1737	18.7	1.9	1.4-2.6	1.5	1.1-2.2	1.3	0.9-1.9	2187	16.5	1.9	1.5-2.5	1.7	1.2-2.4	1.4	1.0-1.9
Rural	1276	10.6	1	-	1	-	1	-	1708	9.3	1	-	1	-	&	6
**Region**	3013	*p* < 0.001			*p* < 0.001		*p* < 0.001		3895	*p* < 0.001			*p* < 0.001		*p* = 0.0026	
Tunis	356	18.4	3.0	1.9-4.6	2.3	1.4-3.7	2.0	1.2-3.3	496	17.3	2.0	1.3-3.1	1.8	1.2-2.9	1.7	1.1-2.5
North East	460	12.2	1.8	1.2-2.8	1.6	1.0-2.5	1.5	1.0-2.3	460	10.4	1.1	0.7-1.7	1.1	0.7-1.7	1.0	0.6-1.5
North West	459	7.1	1	-	1	-	1	-	588	9.6	1	-	1	-	1	-
Centre East	461	24.5	4.2	2.8-6.4	3.9	2.6-6.0	3.5	2.3-5.3	520	18.3	2.1	1.4-3.2	2.0	1.3-3.1	1.8	1.2-2.7
Centre West	495	12.8	1.9	1.1-3.2	2.1	1.2-3.4	2.1	1.3-3.4	613	7.1	0.7	0.5-1.2	0.8	0.5-1.3	0.8	0.5-1.3
South East	392	14.6	2.2	1.4-3.6	2.1	1.3-3.4	1.9	1.1-3.0	604	17.2	2.0	1.3-3.0	1.9	1.2-2.9	1.6	1.1-2.5
South West	390	11.9	1.8	1.1-2.8	1.7	1.1-2.6	1.6	1.0-2.5	614	13.9	1.5	0.9-2.6	1.5	0.9-2.5	1.4	0.8-2.3
**Familial factors**																
**Family history T2D**	3013	*p* < 0.001			*p* < 0.001		*p* < 0.001		3895	*p* < 0.001			*p* < 0.001		*p* < 0.001	
Yes	667	26.3	2.5	2.0-3.1	2.5	1.9-3.1	2.4	1.8-3.0	964	22.3	2.3	1.9-2.9	2.7	2.1-3.4	2.6	2.1-3.3
No	2346	12.5	1	-	1	-	1	-	2931	11.0	1	-	1	-	1	-
**Demographic factors**																
**Age (year)**	3013	*p* < 0.001			*p* < 0.001		*p* < 0.001		3895	*p* < 0.001			*p* < 0.001		*p* < 0.001	
35-39	540	9.2	1	-	1	-	1	-	709	5.1	1	-	1	-	1	-
40-44	661	11.9	1.3	0.8-2.2	1.3	0.8-2.2	1.3	0.8-2.2	758	8.9	1.8	1.1-3.1	1.7	1.0-2.9	1.6	0.9-2.8
45-49	560	17.1	2.0	1.3-3.3	2.1	1.3-3.3	2.2	1.4-3.5	724	12.0	2.5	1.6-4.1	2.3	1.4-3.8	2.2	1.4-3.7
50-54	405	19.2	2.3	1.4-3.9	2.5	1.5-4.1	2.6	1.6-4.4	592	18.4	4.2	2.8-6.4	4.0	2.6-6.1	3.9	2.5-6.0
55-59	287	20.7	2.6	1.5-4.4	2.8	1.6-4.8	2.8	1.6-4.9	415	23.0	5.6	3.4-9.1	5.1	3.0-8.9	5.3	3.1-9.1
60-64	232	24.2	3.1	1.7-5.7	3.8	2.0-7.2	4.2	2.2-8.1	300	22.3	5.4	3.2-8.9	5.0	2.9-8.9	5.1	2.9-9.0
65-70	328	22.0	2.8	1.6-4.7	3.6	2.1-6.3	3.8	2.1-6.7	397	26.4	6.7	4.0-11.1	6.6	3.9-11.4	6.6	3.8-11.4
**Parity**									3895	*p* < 0.001			*p* = 0.41		*p* = 0.30	
0-3	-		-	-	-	-	-	-	1213	11.0	1	-	1	-	1	-
4-5	-		-	-	-	-	-	-	1197	13.3	1.2	0.9-1.6	0.9	0.7-1.3	1.1	0.8-1.4
6+	-		-	-	-	-	-	-	1485	18.3	1.8	1.4-2.4	1.1	0.8-1.6	1.3	0.9-1.8
**Socio-economic factors**																
**Economic level of the household proxy**	3013	*p* < 0.001			*p* = 0.0024		*p* = 0.32		3895	*p* < 0.001			*p* < 0.001		*p* = 0.23	
1st quintile	679	10.5	1	-	1	-	1	-	976	8.5	1	-	1	-	1	-
2nd quintile	654	12.3	1.2	0.8-1.8	1.2	0.8-1.8	1.0	0.7-1.6	957	13.1	1.6	1.2-2.3	1.7	1.2-2.4	1.3	0.9-1.8
3rd quintile	602	16.1	1.6	1.1-2.5	1.6	1.0-2.6	1.4	0.9-2.3	812	17.2	2.2	1.6-3.2	2.3	1.6-3.3	1.5	1.0-2.3
4th quintile	598	17.5	1.8	1.2-2.8	2.0	1.2-3.0	1.4	0.9-2.3	646	16.2	2.1	1.5-2.9	2.2	1.5-3.2	1.3	0.9-2.1
5th quintile	480	22.2	2.4	1.6-3.7	2.5	1.5-4.1	1.7	1.0-3.0	504	15.5	2.0	1.3-3.0	3.2	1.8-5.6	1.9	1.0-3.5
**Level of education of head of household**	3013	*p* = 0.20			*p* = 0.73		*p* = 0.74		3895	*p* < 0.001			*p* = 0.094		*p* = 0.073	
No formal schooling	839	14.7	1	-	1	-	1	-	1708	17.0	1	-	1	-	1	-
Primary school	1258	14.6	1.0	0.6-1.4	1.0	0.6-1.6	0.9	0.5-1.5	1414	12.8	0.7	0.6-0.9	0.8	0.6-1.1	0.8	0.6-1.0
Secondary	678	17.7	1.3	0.9-1.8	0.8	0.5-1.5	0.8	0.4-1.6	586	13.9	0.8	0.6-1.1	0.8	0.5-1.3	0.8	0.5-1.3
University	238	20.4	1.5	0.9-2.4	1.7	0.3-9.2	1.6	0.3-10.0	187	5.9	0.3	0.2-0.6	0.4	0.2-0.8	0.4	0.2-0.8
**Level of education**	3013	*p* = 0.15			*p* = 0.32		*p* = 0.59		3895	*p* = 0.013			*p* = 0.019		*p* = 0.086	
No formal schooling	745	14.7	1	-	1	-	1	-	2253	14.9	1	-	1	-	1	-
Primary school	1214	14.2	1.0	0.7-1.3	1.3	0.7-2.3	1.1	0.6-2.0	1146	15.3	1.0	0.8-1.3	1.6	1.1-2.2	1.3	0.9-1.9
Secondary	802	18.0	1.3	0.9-1.8	1.6	0.8-3.0	1.2	0.6-2.5	411	11.2	0.7	0.5-1.1	1.1	0.7-1.8	0.9	0.5-1.5
University	252	19.4	1.4	0.9-2.3	0.7	0.1-4.1	0.5	0.1-3.5	85	4.2	0.2	0.1-0.7	0.6	0.2-2.4	0.5	0.1-1.9
**Professional activity**^**7**^	2597	*p* = 0.0022							2573	*p* = 0.45						
Not working/retired	87	17.2	1.3	0.6-2.8	-	-	-	-	2044	13.5	1.2	0.8-1.9	-	-	-	-
Employee/Worker	1662	13.8	1	-	-	-	-	-	313	11.4	1	-	-	-	-	-
Intermediate level	428	22.5	1.8	1.3-2.5	-	-	-	-	150	12.8	1.1	0.6-2.3	-	-	-	-
Upper level	420	14.3	1.0	0.7-1.6	-	-	-	-	66	7.2	0.6	0.2-1.8	-	-	-	-

1- Model 0: unadjusted association.

2- Model 1: associations adjusted for demographic (age, parity if women) and socio-economic factors (economic level of the household proxy, education of head of household and subject).

3- Model 2: associations adjusted for demographic and socio-economic factors (same as model 1), familial history of T2D and environmental factors (milieu and region).

4- Weighted % of T2D.

5- Odds-ratio of category to reference category.

6- Confidence interval (P = 0.95).

7- Professionnal activity not included in multivariate models due to high rate of missing data.

For both genders, the increase in prevalence of T2D with age was marked and not confounded by any of the other factors included in the analyses. However, the increase was different according to gender. Either family history of T2D, socio-economic or environmental; the association was stronger for women. Prevalence of diabetes increased with parity but with no independent association once adjusted (Table 3).

A sharp increase of prevalence of T2D with economic level of the household was observed, minimally confounded by other demographic or education level; the marked differences between environments (either area or region) regarding economic level and diabetes was reduced once these variables were included in the models. Education of head of the household was not associated with T2D for men, but a higher level was associated with a decrease for women, though somewhat less markedly once adjusted for socio-demographic factors (e.g. for university degree vs. none education, adjusted [OR (95%), 0.4 (0.2-0.8)]. The individual education level was associated to T2D only for women, with an inverse U-shaped relationship, the higher risk being for those with primary schooling (primary vs. none: adjusted [OR (95%), 1.6 (1.1-2.2)] (Table 3).

There were no marked differences according to region for neither undiagnosed nor untreated T2D. Those with family history of T2D were more aware about their diabetes (adjusted OR = 0.5 [0.3-0.7], *p* < 0.001). Due to smaller sample size of the sub sample of untreated T2D, the inference was to stand by the null hypothesis (adjusted OR = 0.6 [0.3-1.4], *p* = 0.25). The probability of both undiagnosed and untreated T2D decreased with age but only undiagnosed with economic level of the household either adjusted or not. The observed contrast for untreated T2D between urban and rural, increased after adjustment for family history of T2D, socio-demographic variables and region (from 1.3 [0.6-2.8], *p* = 0.51 to 1.8 [0.8-4.3], *p* = 0.18 CR = -28%) (Table 4).

**Table 4:** Association of environmental and socio-demographic variables with undiagnosed and untreated type 2 diabetes (n = 6908)

	**Undiagnosed diabetes**								**Untreated diabetes**							
		**Crude associations**			**Adjusted associations**		**Adjusted associations**				**Crude associations**		**Adjusted associations**		**Adjusted associations**	
		**(Model 0)**^**1**^			**(Model 1)**^**2**^		**(Model 2)**^**3**^				**(Model 0)**^**1**^		**(Model 1)**^**2**^		**(Model 2)**^**3**^	
	**n**	**%**^**4**^	**OR**^**5**^	**C.I.**^**6**^	**OR**^**5**^	**C.I.**^**6**^	**OR**^**5**^	**C.I.**^**6**^	**n**	**n**	**%**^**4**^	**OR**^**5**^	**C.I.**^**6**^	**OR**^**5**^	**C.I.**^**6**^	**OR**^**5**^
**Environment**																
**Milieu**	949	*p* = 0.23			*p* = 0.41		*p* = 0.31		468	*p* = 0.51			*p* = 0.42		*p* = 0.18	
Urban	663	49.4	0.8	0.6-1.2	1.2	0.8-1.7	1.2	0.8-1.8	335	12.4	1.3	0.6-2.8	1.4	0.6-3.5	1.8	0.8-4.3
Rural	286	54.9	1	-	1	-	1	-	133	9.9	1	-	1	-	1	-
**Region**	949	*p* = 0.27			*p* = 0.54		*p* = 0.44		468	*p* = 0.28			*p* = 0.20		*p* = 0.14	
Tunis	149	46.4	0.7	0.4-1.3	0.9	0.5-1.8	0.9	0.4-1.9	83	9.0	0.4	0.1-0.9	0.3	0.1-0.8	0.3	0.1-0.7
North East	105	40.6	0.6	0.3-1.1	0.6	0.3-1.2	0.6	0.3-1.3	60	15.0	0.6	0.2-1.8	0.5	0.2-1.5	0.5	0.2-1.6
North West	92	54.2	1	-	1	-	1	-	41	21.8	1	-	1	-	1	-
Centre East	209	57.4	1.1	0.7-2.0	1.1	0.6-2.1	1.1	0.6-2.2	83	10.6	0.4	0.2-1.2	0.3	0.1-0.9	0.3	0.1-1.0
Centre West	107	53.2	1.0	0.5-1.8	0.8	0.5-1.7	0.8	0.4-1.6	50	16.3	0.7	0.3-2.0	0.7	0.2-2.3	0.7	0.2-2.4
South East	153	50.1	0.9	0.5-1.5	0.9	0.5-1.7	0.9	0.4-1.7	76	12.2	0.5	0.2-1.3	0.4	0.1-1.2	0.4	0.1-1.2
South West	134	44.9	0.7	0.3-1.4	0.7	0.3-1.7	0.7	0.3-1.6	75	7.1	0.3	0.1-0.9	0.2	0.1-1.0	0.2	0.1-0.9
**Familial factors**																
**Family history of T2D**	949	*p* < 0.001			*p* < 0.001		*p* < 0.001		468	*p* = 0.25			*p* = 0.20		*p* = 0.25	
Yes	369	42.1	0.6	0.4-0.7	0.5	0.4-0.7	0.5	0.3-0.7	214	9.8	0.7	0.3-1.3	0.6	0.3-1.3	0.6	0.3-1.4
No	580	56.9	1	-	1	-	1	-	254	13.8	1	-	1	-	1	-
**Demographic factors**																
**Age (year)**	949	*p* < 0.001			*p* < 0.001		*p* < 0.001		468	*p* = 0.0058			*p* = 0.0089		*p* = 0.0067	
35-39	69	77.8	1	-	1	-	1	-	16	36.1	1	-	1	-	1	-
40-44	134	66.2	0.6	0.3-1.2	0.6	0.3-1.2	0.6	0.3-1.3	43	32.0	0.8	0.2-3.2	0.7	0.2-2.5	0.8	0.2-3.0
45-49	165	59.5	0.4	0.2-0.9	0.4	0.2-0.9	0.4	0.2-0.9	66	7.8	0.2	0.0-0.6	0.1	0.0-0.5	0.1	0.0-0.5
50-54	155	38.5	0.2	0.1-0.4	0.2	0.1-0.4	0.2	0.1-0.4	82	7.8	0.2	0.0-0.6	0.1	0.0-0.5	0.2	0.0-0.6
55-59	145	41.3	0.2	0.1-0.4	0.2	0.1-0.5	0.2	0.1-0.5	84	9.8	0.2	0.0-0.8	0.2	0.0-0.7	0.2	0.0-0.7
60-64	116	41.9	0.2	0.1-0.4	0.2	0.1-0.5	0.2	0.1-0.4	69	9.0	0.2	0.0-0.7	0.2	0.0-0.7	0.2	0.0-0.8
65-71	165	36.8	0.2	0.1-0.3	0.2	0.1-0.4	0.2	0.1-0.4	108	6.9	0.1	0.0-0.6	0.1	0.0-0.5	0.1	0.0-0.5
**Socio-economic factors**																
**Economic level of the**																
**household proxy**	949	*p* = 0.0025			*p* < 0.001		*p* < 0.001		468	*p* = 0.30			*p* = 0.25		*p* = 0.33	
1st quintile	141	69.6	1	-	1	-	1	-	51	10.0	1	-	1	-		
2nd quintile	187	57.3	0.6	0.3-1.0	0.5	0.3-0.9	0.6	0.3-1.0	81	9.7	1.0	0.3-3.4	1.3	0.3-4.8	1.3	0.3-5.4
3rd quintile	228	44.9	0.4	0.2-0.6	0.3	0.2-0.5	0.3	0.1-0.5	124	18.3	2.0	0.7-6.2	2.3	0.6-8.7	2.5	0.6-10.3
4th quintile	203	50.5	0.5	0.3-0.8	0.3	0.2-0.6	0.3	0.2-0.6	106	7.5	0.7	0.2-2.4	0.8	0.2-2.9	0.8	0.2-3.7
5th quintile	191	42.5	0.3	0.2-0.6	0.2	0.1-0.4	0.2	0.1-0.4	106	11.7	1.2	0.4-3.8	1.0	0.2-4.1	1.1	0.2-5.4
**Level of education of head of household**	949	*p* = 0.026			*p* = 0.45		*p* = 0.33		468	*p* = 0.13			*p* = 0.93		*p* = 0.93	
No formal schooling	373	45.0	1	-	1	-	1	-	200	7.9	1	-	1	-	1	-
Primary school	335	57.7	1.7	1.1-2.4	1.3	0.8-2.2	1.5	0.9-2.4	151	15.9	2.2	1.0-4.7	1.2	0.4-3.5	1.2	0.4-3.5
Secondary/university	241	48.3	1.1	0.8-1.7	1.0	0.5-2.0	1.2	0.6-2.3	117	12.5	1.7	0.7-3.9	1.1	0.3-4.9	1.1	0.2-5.0
**Level of education**	949	*p* = 0.047			*p* = 0.36		*p* = 0.38		468	*p* = 0.11			*p* = 0.86		*p* = 0.81	
No formal schooling	403	44.4	1	-	1	-	1	-	222	8.0	1	-	1	-	1	-
Primary school	324	55.7	1.6	1.1-2.3	1.1	0.6-1.9	1.0	0.6-1.8	143	16.7	2.3	1.1-4.9	1.3	0.5-3.4	1.4	0.5-4.0
Secondary/university	223	52.0	1.4	0.9-2.0	1.5	0.8-2.8	1.5	0.8-2.7	103	12.1	1.6	0.7-3.8	1.3	0.3-5.2	1.3	0.3-5.7
**Professional activity**^**7**^	676	*p* = 0.014							342	*p* = 0.15						
Not working/retired	275	39.3	0.5	0.3-0.8	-	-	-	-	162	15.9	1.8	0.7-4.4	-	-	-	-
Employee/Worker	237	55.3	1	-	-	-	-	-	106	9.7	1	-	-	-	-	-
Intermediate level	99	55.5	1.0	0.6-1.6	-	-	-	-	45	3.4	0.3	0.1-1.8	-	-	-	-
Upper level	65	46.0	0.7	0.4-1.3	-	-	-	-	29	9.7	1.0	0.2-4.7	-	-	-	-

1- Model 0: unadjusted association.

2- Model 1: associations adjusted for demographic (age) and socio-economic factors (economic level of the household proxy, education of head of household and subject).

3- Model 2: associations adjusted for demographic and socio-economic factors (same as model 1), familial history of diabetes and environmental factors (milieu and region).

4- Weighted % of T2D.

5- Odds-ratio of category to reference category.

6- Confidence interval (P = 0.95).

7- Professional activity not included in multivariate models due to high rate of missing data.

Among treated subjects for which relevant information was available, 90.7% (n = 433) where treated by hypoglycemic drugs either or not in combination with insulin; there was no difference between genders nor between urban & rural areas and few subjects were treated by insulin alone. Drugs were supplied by primary health care centers for 57.7% with a difference according to gender, 48.9% for men vs. 66.0% women (*p* < 0.001) and area, 53.3% on urban area vs. 75.2% on rural one (*p* < 0.001). Among those whose diabetes was diagnosed previous to the survey and for which treatment and relevant information was available (data not shown).

## Discussion

This study provided data on T2D, one of the emerging non communicable diseases in Africa and the Middle East, according to geographical, social, and economical characteristics among adult Tunisian population. The overall prevalence of T2D in Tunisia according to WHO criteria was 15.1%, which included 7.4% previously diagnosed T2D and 7.7% newly diagnosed T2D. Similar to our results, the prevalence of T2D was (16.1%) in Oman [15] and (16.7%) in Qatar [16]. Several studies have reported high rates of prevalence of T2D, such as in Bahrain (25.7%) [17], Saudi Arabia (23.7%) [18], Al Ain, United Arab Emirates (17.1%) [19] But, our figures are quite higher than the prevalence found in other countries in the EMR. In Iran, the overall prevalence of self-reported T2D was 6.9% [20]. In Algeria, the diabetes prevalence was 8.2% [21]. In Cyprus, 10.3% [22]. In Spain, total prevalence of T2D was 13.2% [23], Switzerland (11.5%) [24] and Japan (10.1%) [25]. The comparison of our results with these studies results should be done with caution because of different diagnostic criteria used, different methods adopted, how representative sample are and the varying dates that the studies have been performed.

The present study revealed that the prevalence of T2D and IFG was similar in men and women, which is consistent with findings of previous studies [9,19,26,27]. Worldwide, diabetes occurs equally in men and women, but is slightly higher in men under 60 years of age and in women at older age: the female excess is more pronounced in developed countries [28,29]. Previous reports from Arab countries have shown variables results regarding gender differences in the prevalence of T2D [16,18]. Similar to other studies [16,19,21,27,28], our results demonstrated that T2D increased with age for both genders, though the increase was more pronounced among women than men. Hormonal factors, postmenopausal weight gain, and a different risk profile might account for the higher age-specific prevalence rates of T2D among women compared with men.

The prevalence of T2D was related to urbanization in Tunisia. These findings are in accordance with previous studies showing a higher prevalence of T2D in urban areas, compared to rural parts [9,18,30]. A possible explanation for the higher urban prevalence of T2D in Tunisia could be due to the increasing cardiovascular risk factors in the urban area, due to the changes caused by the epidemiological transition, including increased fat and caloric intake and decreased activity, for an Eastern Mediterranean country.

Tunisia, as most of the Eastern Mediterranean Region countries, is facing a crucial epidemiological transition [5,7]. However, the transition is complex and the contrasts between the regions in Tunisia are mirrored through urbanization, and demographic and socio-economic indicators [30,31]. The coastal regions have achieved a sustainable socioeconomically development while the North West and Centre West have only recently emerged from poverty and are still less developed regions. They do not face the same crucial epidemiological transition than the District of Tunis and the East and the South East, the epicenter of the epidemiological transition in Tunisia. Our findings revealed that the prevalence of T2D varied significantly according to Tunisian regions, being higher in the most developed ones. Previous studies showed that diabetes is patterned by SES with persons of lower SES having higher prevalence and incidence of diabetes [32,33]. According to our study, T2D prevalence was positively associated with economic level of the household for both men and women and inversely associated with education in women. With respect to occupation, no association was found between this variable and T2D in both genders. In our study, for both genders those with a family history of T2D were much more at risk of T2D than those without, which is consistent with other results. Family history information may serve as a unique and useful tool for public health and prevention medicine [34].

Another important finding from this study is the low detection rate of T2D almost 50% of diabetes was undiagnosed, which is similar to the figure in Algeria (50%) [21], Spain (48%) [23], and Australia (47%) [35]. But our study rate is considerably higher than the reported rate in Sultanate of Oman that only one third of Omani diabetic subjects knew that they had diabetes [15] and in Qatar (35.5%) [16]. The rate remains lower than that in Danish (70%) [36] and Indians (60.6%) [37]. In our population study, awareness of diabetes tended to increase with age in both genders. Several explanations for this increase with age can be considered. The T2D screening among adults have recently introduced in the public health programs, is indicated to people since 40 years old at high risk, the (obese, family history of diabetes and other risk factors).

There was no difference between rural and urban areas and regions the accessibility of health facilities explain the higher level of diabetes diagnosis and treatment. More than two third of women and a half of men obtain their drugs only from the Primary Health Care Centers HC. However, in these centers, the drugs are limited to one or two old classes and the PHC are often out of stock.

Our study has strengths and limitations. The strengths include the large sample consisting of both urban and rural populations; a sound representation of the national population, and detailed information on potential confounding factors. As a cross-sectional study, the present analysis however, limited in its ability to elucidate a causal relationship. This limitation also prevents any measure of temporal changes in prevalence of T2D and factors associated with T2D. Longitudinal studies would complement the present study to determine causality and directional effect of the factors.

## Conclusion

The prevalence of diabetes in Tunisia is high and it confirmed that so far controlling transmitted diseases seems to be successful, Tunisian people are about to face new problems as diabetes. The increase in prevalence is primarily being driven by environmental factors, nutritional transition and westernization of the lifestyle. The age distribution of the population and the growing prevalence of obesity are alarming signs that the worst is still to come. Well planned strategies urgently needed to reduce the burden of diabetes. An enormous effort is required to educate the population to modify the lifestyle and to train physician to improve the diagnosis and treatment of patients with diabetes and managers for enhancing the availability of efficient drugs.

## Competing interests

The authors declare that they have no competing interests.

## Authors’ contributions

HBR conceived the protocol, managed data collection, drafted and finalized the manuscript; SBA researched literature, contributed to interpretation of the results and to writing the manuscript; PT contributed to the study design and conducted statistical analyses, HS and SB contributed to the conception and design of data; BM, FD and NA contributed to the study design. All authors reviewed and edited the manuscript and approved the final version of the manuscript.

## Pre-publication history

The pre-publication history for this paper can be accessed here:

http://www.biomedcentral.com/1471-2458/14/86/prepub
